# Copy number variation in the Framingham Heart Study

**DOI:** 10.1186/1753-6561-3-s7-s133

**Published:** 2009-12-15

**Authors:** Corina Shtir, Roger Pique-Regi, Kim Siegmund, John Morrison, Fredrick Schumacher, Paul Marjoram

**Affiliations:** 1 Department of Preventive Medicine, Keck School of Medicine, University of Southern California, 1540 Alcazar Street, CHP 220, Los Angeles, California 90089, USA; 2 Department of Electrical Engineering, Viterbi School of Engineering, University of Southern California, 3650 McClintock Avenue, Los Angeles, California 90089, USA; 3 Department of Pediatrics, Children's Hospital Los Angeles, Keck School of Medicine, University of Southern California, 4650 Sunset Boulevard, Los Angeles, California 90027, USA

## Abstract

In this paper we test for association between copy number variation and diabetes in a subset of individuals from the Framingham Heart Study. We used the 500 k SNP data and called copy number variation using two algorithms: the genome alteration detection algorithm of Pique-Regi et al. and the software Golden Helix. We then tested for association between copy number and diabetes using a gene-based analysis. Our results show little evidence of association between copy number and diabetes status. Furthermore, our results indicate a relatively poor level of agreement between copy number calls resulting from the two programs. We then examined potential causes for this difference in results and the implications for future studies.

## Background

One of the most important challenges facing the field of biology today is making sense of genetic variation. One form of variation that is gaining an increasing level of attention is copy number variation (CNV). The term CNV is used to encompass a variety of polymorphisms that occur at the sequence level and that affect the copy number of regions of genetic material. Examples include insertions, deletions, rearrangements, and duplications. While humans have CNV, the degree of that CNV is only now becoming appreciated [1,2], and its influences on phenotypic variation is not yet well understood. However, while a number of associations between single-nucleotide polymorphisms (SNPs) and disease have been verified, it remains the case that the effect sizes of these polymorphisms appear relatively small, with reported odds ratios typically being below 1.5 (see Estivill and Armengol [3] for a recent review). While the reasons for this remain unclear, it is plausible that SNP data are not capturing all the relevant associations. Consequently, in this paper we implement population-based tests for association between CNV and phenotypic variation within the Framingham Heart Study samples provided as part of Genetic Analysis Workshop 16 Problem 2.

## Methods

### Copy number inference

CNV inference algorithms start by analyzing 'intensities' for each probe at each SNP location. Intensities are generated from .cel files via the Affymetrix Power Tools software [4]. Our major focus is then on two CNV-calling methods: 1) the genome alteration detection algorithm (GADA) of Pique-Regi et al. [5], and 2) the HelixTree copy-number analysis module (CNAM) [6]. Both methods begin by normalizing intensities for SNPs across all chips. Such normalization is designed to correct for problems such as batch effects (the intensities for a sample may depend globally upon the batch in which the sample was analyzed) or local defects on the genotyping hardware. The CNAM analysis uses a proprietary normalization scheme for which details are not available. The GADA analysis employs a median normalization step [5] followed by the widely used normalization scheme within the Affymetrix Power Tools suite [4].

GADA copy number analysis employs three main steps: 1) it uses a compact linear algebra representation for the genome copy number from normalized probe intensities, 2) it applies a sparse Bayesian learning technique, and 3) it uses a backward-elimination procedure that ranks the inferred points from the previous step and also efficiently adjusts the accuracy trade-off between sensitivity and false-discovery rate. Pique-Regi et al. [6] tested their method on a variety of actual and simulated data sets and concluded that it achieved the highest accuracy, lowest false-discovery rate, and was, by several orders of magnitude, faster than competing methods.

CNAM employs an optimal segmenting algorithm that searches regions of markers in which log_2 _ratios vary significantly from region to region and therefore confer variation in copy number. Log_2 _ratios are created by normalizing raw intensity data against a reference sample. The CNAM segmenting process is optimized through 1) subdivision of the chromosomal region of markers into a moving window of sub-regions and 2) a permutation algorithm that validates the found cut-points. We used a moving window of 10,000 markers. One thousand permutations were used to assess significance.

### Association between variation in copy number and phenotype

One of the central motivations for better understanding patterns of CNV is to then relate those patterns to phenotypic variation. Such applications are in their infancy, and in this paper we propose a relatively straightforward technique for addressing this issue. The output from the CNV calling algorithms we employ here can be regarded as a piece-wise constant function that indicates the estimated number of copies of each allele for each individual over the entire length of the genome. Changes in state of this function correspond to changes in (estimated) copy number. The points at which these changes in copy number occur are likely to be correlated across individuals, due to shared ancestry, but will certainly still vary across individuals. Thus, when summarizing the output of such an algorithm across an entire sample of interest, we break the genome up into a set of intervals, ***I ***= {***I***_*j*_}, ***j ***= 1, ..., ***n***, such that the copy number does not *change for any individual in the sample *within any interval. For convenience, we define intervals to start and end at locations corresponding to the midpoint between neighboring SNPs. Clearly, it is most sensible to use a set *I *that contains the minimal number of intervals, which means that the end of an interval will correspond to a point at which estimated copy number changes for at least one member of the sample.

Our analysis method focuses directly on genes. For a given gene we calculate the mean copy number across all segments contained within that gene for each individual. Because estimated copy number is not obviously normally distributed, we then test for a difference in the rank of estimated copy number between cases and controls using a Wilcoxon rank-sum test. This results in a *p*-value for each gene.

## Results

Our analysis currently focuses on a subset of Offspring Cohort individuals with diabetes. In order to protect against undiagnosed diabetes, we insisted that cases had a fasting plasma glucose measure of at least 126 mg/dl, while controls had a fasting measure less than 110 mg/dl. The controls were also frequency-matched to cases by 5-year age intervals, sex, and ever smoked status (age and smoking history taken at baseline). Some individuals are related, but the majority are not (at least by the pedigree information provided). This resulted in a final sample size of 194 cases and 213 controls, for which we analyzed the 500 k SNP data. We then called copy number. Space prohibits a display of estimated copy number here, but, in summary, we observed that previously known sites of copy number variations [2] were detected as such in this data set, and that, for each method, there is clear correlation between the locations at which copy number changes across individuals (despite the fact that the algorithm call copy number independently for each individual). As expected, the number of changes of copy number in a gene was directly proportional to its length (correlation coefficient = 0.89).

**Figure 1:**
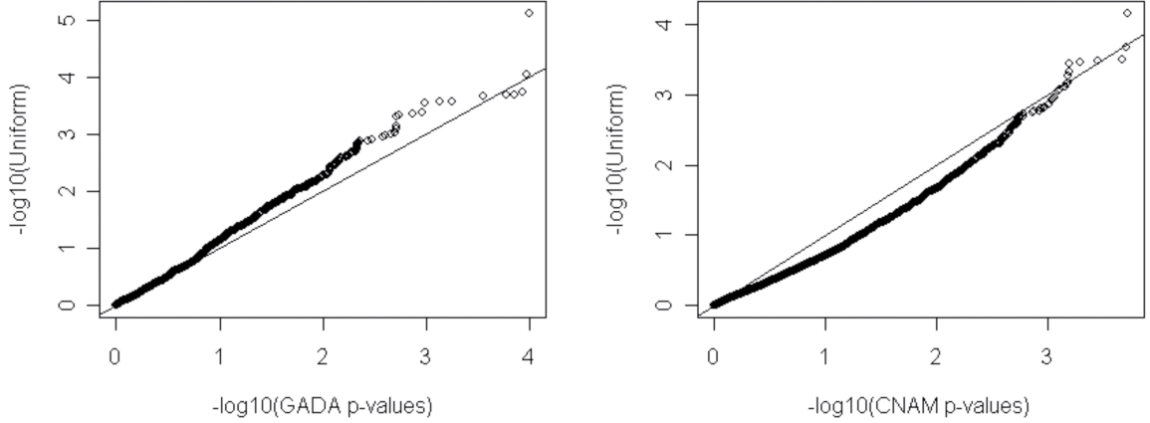
**Q-Q Plot of *p*-values for gene-based average CNV associations for GADA and CNAM**. The plot shows observed (x-axis) and expected (y-axis) values of -log_10 _*p*-values resulting from the gene-based test for association between CNV and diabetes using the GADA and CNAM analyses.

**Figure 2:**
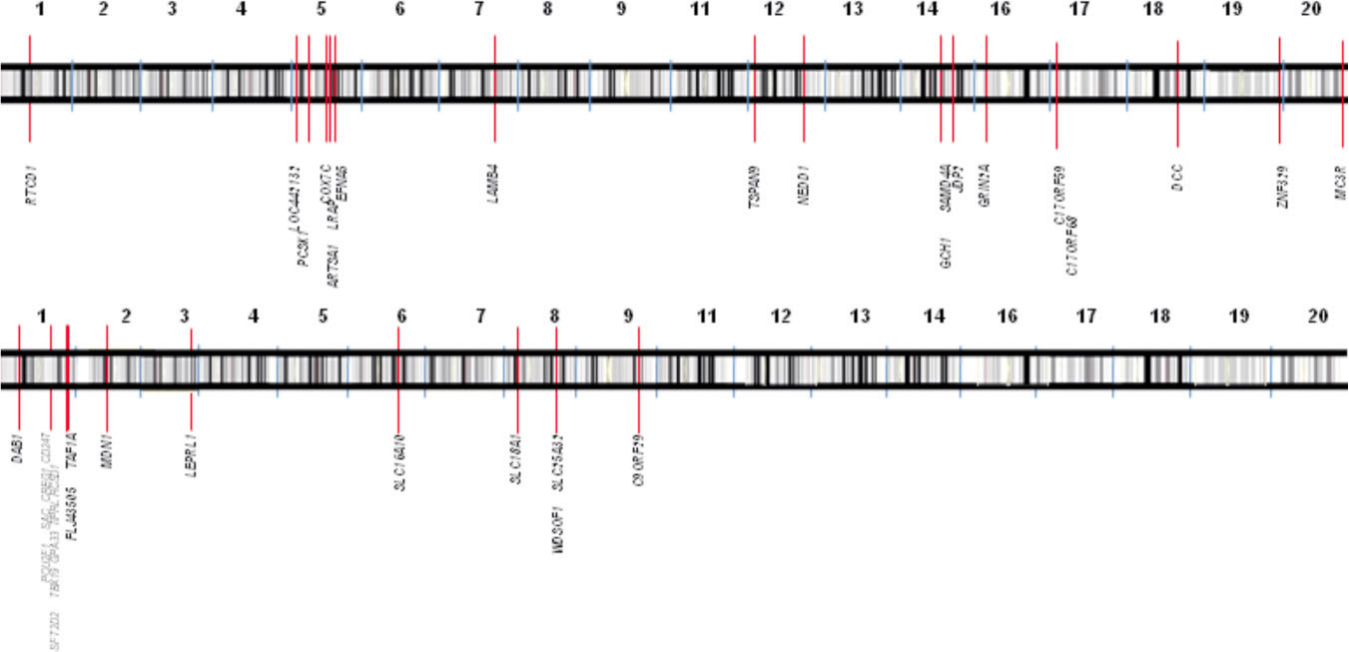
**Top 20 genes with most significant CNV associations. **We show the distribution of potentially significant genes varies along the genome. The upper plot shows results from the GADA gene-based analysis; the lower shows those from the CNAM analysis. In each case, red bars show the 20 genes that have the smallest *p*-value when testing association with diabetes.

**Figure 3:**
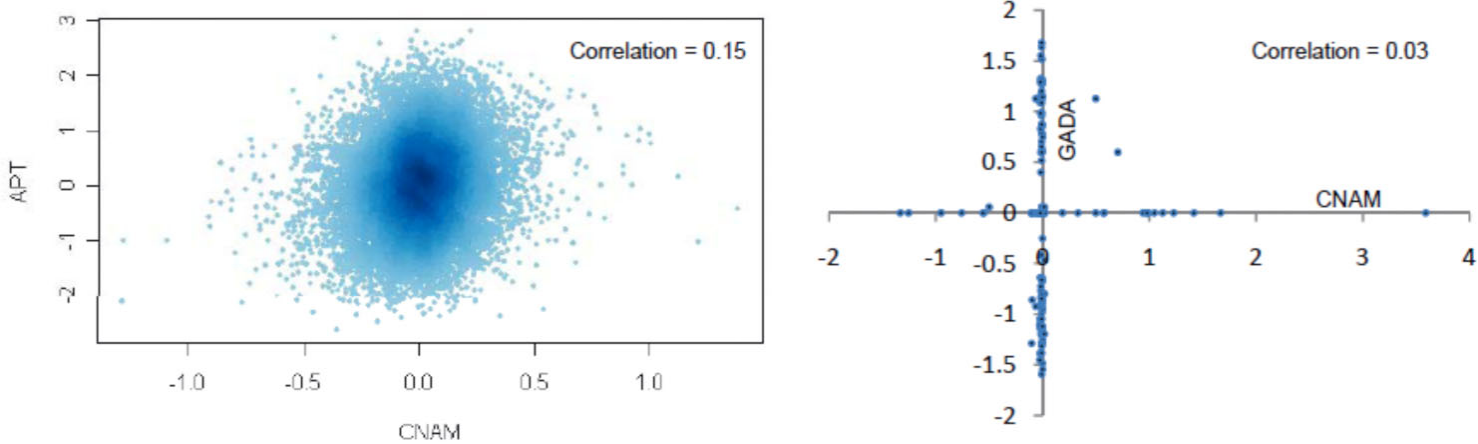
**Scatter plots**. The left plot shows a heat-map of normalized SNP intensities resulting from the CNAM normalization (x-axis) and APT normalization used for the GADA analysis (y-axis) for a randomly chosen individual. There is a striking lack of correlation between the results of the two normalization routines. The right plot is a scatter plot of resulting copy number calls for randomly chosen (but representative) regions along the genome. Again, we note a striking lack of agreement between calls resulting from CNAM and GADA.

Table 1 presents a summary of the results for the GADA and CNAM analyses for each chromosome. No gene attains genome-wide significance when tested for association between copy number and diabetes. However, some interesting features are observed. For example, under the null hypothesis of no association, *p*-values should be uniformly distributed, with each gene having a probability of 0.05 of being reported as 'significant' on this basis. Thus, for each chromosome, the number of genes showing association at the level *p *< 0.05 will be binomially distributed. We used this to test whether there was an over- or under-representation of such associated genes on any chromosome. After a multiple comparison correction, several chromosomes showed evidence of excess, or lack, of associated genes. Furthermore, there is a clear excess of small *p*-values for the CNAM analysis. This can be seen in Figure 1, where we show a quartile-quartile (Q-Q) plot of observed vs. expected *p*-values genome-wide (we plot -log_10 _of the *p*-values). The Q-Q plots reflect the relative over- or under-abundance of small *p*-values resulting from the CNAM or GADA analysis. In Figure 2 we show how the distribution of the genes with the smallest *p*-values when tested for association with diabetes varies along the genome for the GADA and CNAM analyses. There appears to be little agreement between the two methods. This raises the question of why the results of the methods show such poor agreement among these genes. Recall that the first step for both methods is the normalization of SNP intensities, but that the two methods employ different normalization techniques. We therefore examined the degree of agreement between the SNP intensities after normalization. We show this in Figure 3 (left side), a scatter plot of the intensities resulting from the two schemes. The results are striking: there is very poor correlation between the normalized intensities. Thus, even if the methods were using identical routines to call copy number, which they are not, we would expect to obtain widely different calls of copy number, with consequent large differences between *p*-values resulting from subsequent tests of association between copy number and phenotype. We show the poor agreement in copy number in Figure 3 (right side). It is important to note that both methods use somewhat arbitrary definitions of upper and lower cut-points (CU and CL, respectively), defined such that if the normalized intensity for a SNP is below CL it is determined to have a copy number less than two, whereas if normalized intensity is above CU the copy number is called as greater than two; otherwise the copy number is called as two. These arbitrary cut-points are different for the two methods and, combined with the differing results from normalization, result in wildly different calls of copy number. Most often both methods call copy number as two, but it is seldom the case that the methods simultaneously determine copy number to be other than two for any given SNP. This, clearly, is a concern.

**Table 1:** Summary of gene-based CNV associations for GADA based segmentation (Wilcoxon rank-sum test)

			% Significant genes^c^	
			
Chr	Affymetrix genes^a^	Genotyped genes^b^	GADA	CNAM
1	1650	1500	5.12	12.60
2	1046	974	0.72	16.32
3	893	829	0.36	13.51
4	661	634	1.31	1.57
5	722	680	7.45	22.35
6	883	822	1.84	18.86
7	709	655	3.09	6.87
8	539	505	10.18	1.78
9	632	582	0.87	4.81
10	636	603	1.84	8.96
11	1028	907	1.68	4.85
12	871	799	4.80	15.14
13	295	287	8.81	10.45
14	527	474	4.25	12.66
15	507	474	1.92	9.92
16	580	483	5.64	3.52
17	851	712	8.09	0.14
18	241	241	4.17	1.66
19	912	713	1.51	0.14
20	470	439	2.75	5.47
21	201	187	0	9.09
22	365	326	3.12	0
				
Total/Average	15219	13826	3.61	8.62

^a^Overall number of genes as defined by Affymetrix annotation files

^b^Number of those genes for which we had SNP intensity data

^c^Percentage of those genes in which associated CNV was detected at the *p *= 0.05 value (uncorrected for multiple comparisons) using GADA and CNAM

## Conclusion

The importance of CNV has only recently become appreciated. The relationship of CNV to phenotypic variation is even less well developed. While we hope the analysis presented in this paper is a useful step forward in this area, it merely scratches the surface of what is likely to be an extremely complex challenge. CNV lacks some of the 'neatness' of SNP data. It does not occur at well defined positions (i.e., the points at which CNV changes is often different across individuals). Furthermore, for a variety of reasons, the 500 k chip platforms are not ideal for detecting CNV when compared with more recent platforms. It is also the case that many functional mutations occur outside genes (in promoter regions, for example). As such, many regions of CNV may not be detected. It is also likely to be challenging to detect small CNVs using these technologies.

An approach in which we break the genome into regions of maximal length, such that copy number remains constant for each individual within each region, results in an extremely large number of regions (around 82,000 regions for the samples analyzed in this paper). While each region can be treated as if it were a (multi-allelic) locus, and marginal tests can then be performed, such tests are likely to be far from optimal.

The principal reason for this is the correlation between intervals. This is directly analogous to the situation with SNP analysis, but may well be even more complex in this new setting. An alternative approach might be to attempt to relate SNP variation to CNV, and adopt an approach akin to the tag-SNP idea.

In this paper we attempted to move past the perils of a strategy based upon marginal tests by conducting a gene-at-a-time analysis, relating mean copy number within a gene to phenotype. Such an approach seems reasonable, succeeds in reducing the analysis to a reasonable number of tests, and thereby avoids the worst excesses of multiple comparison corrections. However, it should be noted that the very poor agreement in results from the two methods explored in this paper indicates that substantial work remains to be done. It is this lack of agreement that represents the principal lesson to be drawn from the current study. Normalization is regarded as an important, but somewhat routine step in analyses such as these. However, our paper demonstrates that the particular method of normalization chosen can have a key influence on the results obtained. In our case, the two normalization methods are both widely used, and appear inherently sensible, but result in normalized intensities that are very poorly correlated across methods. Consequently, subsequent analyses will produce wildly different results. As the phrase "garbage in, garbage out" reminds us, it is important to ensure that such normalization routines are adding signal, rather than noise to the data. As such, there is an urgent need for a widespread comparison of normalization methods in order to better assess which of them is most effective.

Finally, it should also be noted that, while we look at all genes in the present study, there is little reason *a priori *to expect candidate genes chosen on the basis of a SNP study to also have a function due to CNV. It is entirely possible that genes that affect phenotype through CNV will be distinct from those that have an effect due to SNP polymorphism. In a recent study, Stranger et al. examined data from Phase 1 of HapMap and noted that SNPs and CNVs captured 83.6% and 17.7%, respectively, of the total detected genetic variation in the expression of around 15,000 genes, but that "the signals from the two types of variation had little overlap" [7]. Other studies take a different view (e.g., McCarroll et al. [8]).

## List of abbreviations used

CNAM: Copy-number analysis module; CNV: Copy-number variation; GADA: Genome alteration detection algorithm; Q-Q: Quantile-quartile; SNP: Single-nucleotide polymorphism.

## Competing interests

The authors declare that they have no competing interests.

## Authors' contributions

CS, RPR, KS, RJ and FS developed the methodology and performed the analysis. PM conceived the study. PM and CS wrote the paper.
